# Computationally Modeling Lipid Metabolism and Aging: A Mini-review

**DOI:** 10.1016/j.csbj.2014.11.006

**Published:** 2014-11-15

**Authors:** Mark T. Mc Auley, Kathleen M. Mooney

**Affiliations:** a Faculty of Science and Engineering, Department of Chemical Engineering, Thornton Science Park, University of Chester, UK; b Faculty of Health and Social Care, Edge Hill University, Ormskirk, Lancashire, UK

**Keywords:** Aging, Lipid metabolism, Computational modeling, Deterministic model, Stochastic model, Parameter inference

## Abstract

One of the greatest challenges in biology is to improve the understanding of the mechanisms which underpin aging and how these affect health. The need to better understand aging is amplified by demographic changes, which have caused a gradual increase in the global population of older people. Aging western populations have resulted in a rise in the prevalence of age-related pathologies. Of these diseases, cardiovascular disease is the most common underlying condition in older people. The dysregulation of lipid metabolism due to aging impinges significantly on cardiovascular health. However, the multifaceted nature of lipid metabolism and the complexities of its interaction with aging make it challenging to understand by conventional means. To address this challenge computational modeling, a key component of the systems biology paradigm is being used to study the dynamics of lipid metabolism. This mini-review briefly outlines the key regulators of lipid metabolism, their dysregulation, and how computational modeling is being used to gain an increased insight into this system.

## 1 What is Aging and why Study it Using Computational Systems Biology?

The progress of biomedical science, together with improvements in nutrition and public heath interventions has resulted in a remarkable demographic shift in favor of older people. It is projected that by 2050, 25% of the world's population will be over 60 years of age [1]. There has also been a staggering rise within western populations of those individuals over 85 years of age. For example, in the UK it is predicted that by 2050 almost 5% of the population will comprise of these individuals that are referred to as the ‘oldest old’ [2]. Despite such a dramatic change in population demographics, gerontology remains devoid of a reasonable explanation as to what aging is. Irrespective of this ambiguity, aging is generally recognized as a process that gradually results in the progressive decline of an organism over-time which results in its eventual mortality [3], [4]. The fact that aging comprises every facet of a biological system makes it an inherently difficult phenomenon to study. Therefore the question arises why study aging? If populations are living longer is there a necessity? The answer is that although we are living longer, the extra few years that are gained are not necessarily spent in optimum health. For example, in the UK 37.1% of individuals ≥ 85 years have underlying cardiovascular disease (CVD), which hampers their quality of life [5]. For this reason it is imperative to gain a mechanistic understanding of the impact of aging on the regulation of biological systems. Historically investigating the potential mechanisms which underpin aging has been constrained by their complex and multifaceted nature [6]. This has resulted in aging being studied in a reductionist manner. Fortunately, the last decade and a half has witnessed the growth of systems biology, a discipline which is grounded in understanding biology from an integrated perspective [7], [8]. Dynamic computational modeling resides firmly within the systems biology paradigm [9], [10], [11]. The impact computational modeling is having on lipid metabolism, a system that offers a potential avenue for extending healthy aging will be the focus of this mini-review.

## 2 Lipid Metabolism and Healthy Aging

Aging is underpinned by changes to a number of complex biological mechanisms [12]. Consequently, it is necessary to investigate this phenomenon holistically and where possible examine the impact of age-related changes to biological systems in an interconnected fashion [13]. Of the diseases associated with old age, CVD remains the most prevalent cause of morbidity among older people [14], [15]. The dysregulation of lipid metabolism is known to impact several parameters of cardiovascular health [16]. For instance, there is a well-established relationship between elevated levels of low-density lipoprotein cholesterol (LDL-C) and risk of CVD [17], [18], [19]. Elevated levels of plasma triglycerides (TGs) have also been suggested as a risk factor for CVD, although their association remains a controversial one [20]. Intriguingly, it has also been revealed that certain long lived individuals have a ‘finely-tuned’ lipid profile which may have helped them avoid CVD [21]. From a nutrition perspective adult western diets contain between 30 and 40% of energy from lipids, of which 92–96% are long chain TGs [22]. TGs are composed of a unit of glycerol together with three fatty acids [23]. Long chain TGs are classified on the basis of the number and type of chemical bonds they possess [23]. There are three general classes, saturated fatty acids (SFAs) which have no double bonds, monounsaturated fatty acids (MUFAs) which have one double bond and polyunsaturated fatty acids (PUFAs) which contain two or more double bonds [24], [25]. SFAs are usually solid at room temperature and are mainly found in animal sources such as cream, butter, cheese, milk and animal fats [22]. The main fatty acid in MUFA is oleic acid which is found in olive oil, canola and peanuts [26]. The most ubiquitous PUFA is linoleic acid; its double bonds are in the omega n-6 position. Linoleic acid is found mainly in seed oils, such as sunflower and corn oils [27].

The metabolic fate of dietary lipids can significantly impact health. When SFAs predominate in the diet they tend to raise plasma LDL-C [28]. It is suggested that SFAs increase LDL-C by suppressing the activity and reducing the number of LDL-receptors [29], [30]; the mechanism responsible for the removal of LDL from the circulation [31], [32], [33]. It is also suggested that SFAs cause an increase in the synthesis of LDL-C [34], [35]. Conversely, MUFAs and PUFAs decrease plasma LDL-C levels [36], [37] with evidence indicating that they increase hepatic LDL receptor number and LDL turnover [32]. Thus, the metabolic impact of dietary fats can be viewed as a combination of SFAs, MUFAs and PUFAs [38], [39]. In terms of the metabolic impact of dietary cholesterol, there has been considerable debate as to its effect on plasma cholesterol. For example, dietary cholesterol intake has been shown to result in increases of LDL-C levels even when dietary SFAs are maintained at a low level [40]. On the other hand meta-analysis studies have shown that the effect of dietary cholesterol on plasma cholesterol levels is negligible [41], [42]. In addition dietary cholesterol has only a minimal effect on endogenous cholesterol synthesis, as it is thought whole-body balance is mainly regulated via intestinal absorption [43]. Moreover, studies which monitored plasma cholesterol levels have shown that individuals vary significantly in their responses to dietary cholesterol [44], [45], possibly due to intrinsic differences in cholesterol absorption [46] and have resulted in normolipidemic individuals being classified as either hypo or hyper responsders [47], [48]. Similarly, it has recently been suggested that certain individuals are hyperproducers of cholesterol while others are hyperabsorbers [49]. These findings endorse the view that regulation of whole-body lipid metabolism is underpinned by a variety of extrinsic and intrinsic factors acting simultaneously. It is therefore logical to investigate lipid metabolism using a whole-body framework which is capable of both representing and exploring individual differences in these factors.

**Fig. 1:**
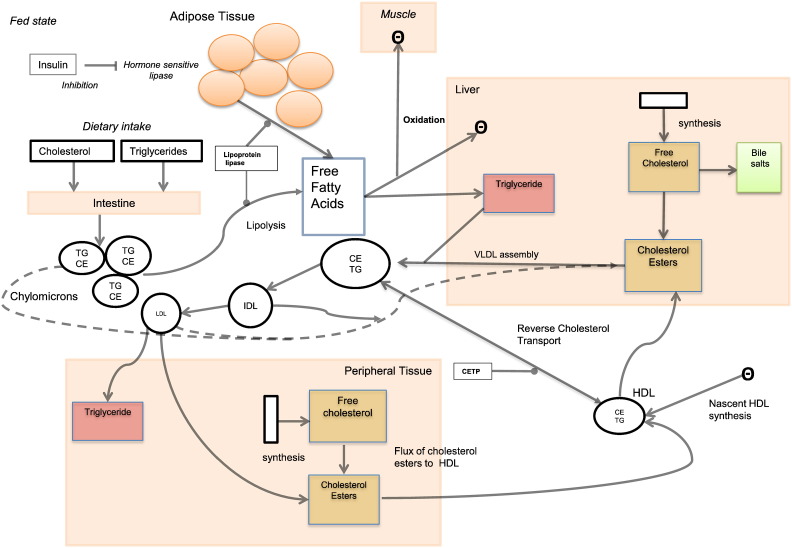
A coarse grained overview of the dynamics of lipid metabolism. The mechanisms outlined in Fig. 1 are discussed in detail in the main body of the article. The Greek letter theta represents utilization, inhibition is represented by an arrow with a flat head, enzymatic activity is represented by rounded headed arrows and conversion or synthesis is represented by conventional arrow heads. Changes to any of the components can have a dramatic impact on lipid metabolism and health.

A number of processes are responsible for maintaining lipid metabolism and changes to any of these mechanisms can disrupt its dynamics, for example as eluded in the previous section whole-body cholesterol balance can be sensitive to changes in cholesterol absorption [50], synthesis [51] and ingestion [52]. Fig. 1 presents a coarse grained overview of the key components of whole-body lipid metabolism. The diagram commences with the dietary intake of lipids. Due to the insolubility of lipids, specialized carriers known as lipoproteins are required to transport them throughout the body [53]. After digestion lipids are taken up by enterocytes and packaged in a lipoprotein called a chylomicron [54], [55]. Absorbed chylomicrons are acted on by lipoprotein lipase (LPL) which hydrolyzes TG releasing free fatty acids (FFAs) and glycerol [56]. The chylomicron remnants are removed hepatically and the FFAs are taken up by muscle and liver for either oxidation or re-esterification [57]. Hormone sensitive lipase (HSL) acts on adipose tissue to release FFAs during lipolysis [58], a process suppressed in fat cells by insulin during the fed state [59]. Hepatic TGs together with cholesterol can be released as part of very low density lipoproteins (VLDLs) [60]. VLDLs are hydrolyzed by LPL, forming VLDL remnants and intermediate density lipoproteins (IDLs). IDLs are either taken up by the liver or hydrolyzed to LDLs, the main cholesterol transporter [60]. LDL is taken up by the liver or by peripheral cells, either independently [61], [62], [63] or via the LDL-receptor [64], [65]. Reverse cholesterol transport (RCT) is the only route for removal of excess cholesterol from peripheral tissue [66]. Crucial to RCT are high density lipoproteins (HDLs) which uptake cholesterol in the peripheral tissue to become high density lipoprotein cholesterol (HDL-C), the so-called ‘good cholesterol’. The excess cholesterol is transferred from the peripheral tissue to the liver where hepatocytes take up the cholesterol and excrete it into the bile either as free cholesterol or as bile salts after conversion [67]. Subsequently, cholesterol and bile salts end up in the feces. Fundamental to RCT is the transport of free cholesterol and phospholipids to lipid-free apo A-I in a process mediated by ATP-binding cassette transporter (ABCA1) [68]. The intimate relationship between ABCA1 and apo A-I leads to the lipidation of apo A-I and generation of a discoid nascent pre-β HDL particle [69], [70]. Following this initial event in RCT, the nascent HDL then matures through the esterification of cholesterol which is mediated enzymatically by lecithin-cholesterol acyltransferase (LCAT) [71]. Mature HDLs can transfer cholesterol directly to the liver in a process mediated by scavenger receptor class B, type I (SR-BI) or alternatively it can transfer cholesterol indirectly by using cholesteryl ester transfer protein (CETP) to reallocate cholesterol to other lipoproteins including LDL and VLDL [72].

## 3 The Impact of Aging and Genetic Variability on the Dynamics of Lipid Metabolism

It is known that plasma lipids increase with age in both males and females [73]. Although the underlying reasons for this rise are not completely delineated, several putative mechanisms have been proposed. For example, in rodents TG absorption can be impaired by as much as 50% with advancing age [74]. This could be due to a defect in lipoprotein assembly or due to a decline in the abundance of fatty acid-binding proteins [74], [75]. Moreover the decrease in TG absorption could be underpinned by a decline in the secretion rate of the key digestive enzyme pancreatic lipase [76]. Conversely, outbred CD-1 mice have demonstrated that intestinal cholesterol absorption efficiency can increase significantly during aging, with 41% of intestinal cholesterol absorbed in old mice compared to 25% in young mice [77]. A gradual reduction in the receptor-mediated clearance of plasma low density LDL-C has also been observed in healthy human males [78], [79] and in rodents [80] with increasing age. This is perhaps a result of a decrease in the number of hepatic LDL receptors [81]. This is consolidated by the finding that old rats can have up to 57% less LDL receptors than young rats [80]. In terms of fatty acid metabolism it has been demonstrated that fatty acid oxidation is impaired in aged muscle during basal conditions, which has been suggested to contribute to TG accumulation with age [82]. Several of the major enzymes involved in lipid metabolism undergo a dramatic change to their behavior with age. For instance, the activity of LPL has been reported to reduce by as much as 55–60% [83], [84], [85]. Inhibition of LPL activity is likely to result in hypertriglyceridemia [86]. It has also been suggested that LPL activity is altered by age-related changes to a broad range of hormones, including cortisol, epinephrine, norepinephrine and leptin (reviewed in [16]). In rodents HSL can suffer a 24% decline in its activity due to age [82]. RCT also suffers from an age-related decline that is suggested to have a ripple effect on the other components of cholesterol metabolism [87]. The decline in RCT could be induced by alterations to CETP, as polymorphisms in the promoter region of the CETP gene have recently been implicated in longevity [88]. Therefore it can be tentatively suggested that CETP is a hub for the regulation of lipid metabolism and even subtle alterations to it could result in dyslipidemia and impact cardiovascular health. It remains unknown exactly how many other genes and single nucleotide polymorphisms (SNPs) influence the dynamics of lipid metabolism and how these can be differentiated from intrinsic aging. One polymorphism that is known to significantly impact lipid metabolism and longevity is that of Apolipoprotein E (apo E) [89], [90]. apo E is a protein associated with chylomicrons, VLDL, and HDL cholesterol. It acts as a ligand for the LDL receptor. The apo E locus has 3 common alleles, E4, E3, and E2 [91]. apo E deficiency results in high levels of cholesterol-enriched lipoproteins, with apo E 4 variant having the most significant impact on lipid metabolism due to its association with elevated LDL-C [92]. Other studies have hinted at as yet undiscovered polymorphisms that could affect lipid metabolism. For example, it has been demonstrated that individuals (Ashkenazi Jewish centenarians) with exceptional longevity and their offspring have significantly larger HDL and LDL particle sizes, indicating that this phenotype could be protective against cardiovascular disease [93].

## 4 The Role Computational Modeling can Play in Understanding Lipid Metabolism

**Fig. 2:**
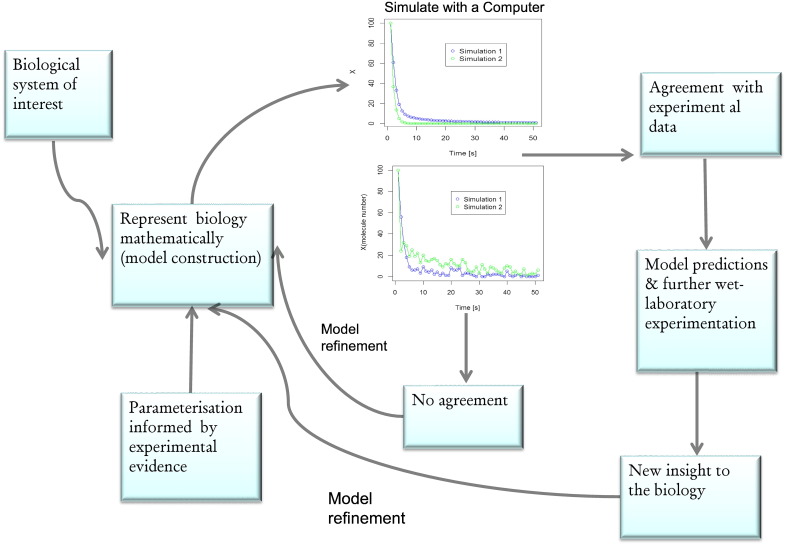
The generic steps involved in constructing a computational systems model and how the process dovetails with experimental work. It is a cyclical process whereby a biological process of interest is identified and then represented mathematically; usually with ordinary differentiation equations. The parameters of the model are informed by current experimental knowledge. These are generally kinetic rate constants. The model is then simulated on a computer and a decision made whether to accept its output or to further refine it. Model prediction can inform further experimental work, which leads to further model refinement and the cycle continues (see also reference [149]).

Computational models have been used for decades in biological research to help understand the dynamics of complex phenomena [94], [95], [96]. Constructing a model requires an intimate knowledge of the system of interest, a hypothesis to test using the model and experimental data to both inform model buildings and to validate the model [9], [97]. Due to the increasing use of the systems biology paradigm there is now an abundance of data that can be used to inform model building [98]. For example, the systems biology approach has resulted in the generation of a diverse array of data which includes metabolic profiles of a variety of organisms under a range of conditions [99], [100]. As a consequence of this ‘new’ data novel computational models can be developed that more fully describe the biochemical reactions within a biological system [101]. The goal of a computer model is to both qualitatively and quantitatively represent the architecture of a biochemical system and to consider the interactivity and dynamics of the various biological entities within the system. Mathematics underpins the reactions within the model with equations used to describe the dynamics of the biochemical mechanisms (reviewed in [97]). There are several different mathematical frameworks that can be employed (reviewed in [97]). The most ubiquitously used framework involves using a set of coupled ordinary differential equations (ODEs) [102]. This was emphasized by Hübner et al. (2011) when over 300 hundred computational systems biology models were surveyed and summarized over a ten year period, with the majority of models found to be ODE based [103]. The ODE framework assumes that the variables behave in a continuous and deterministic manner and makes the assumption that variability is not an important consideration for the model. If on the other hand it is known that variability is an inherent feature of the biological system under consideration, a stochastic model is used. An example of a biological process that is intrinsically stochastic is gene expression [104]. This phenomenon is routinely captured using this modeling approach [105]. The use of stochastic modeling to study lipid metabolism has to date been limited and the majority of models utilize the ODE approach. The reason for this is that in the main models of lipid metabolism have focused on lipoprotein dynamics, whole body cholesterol metabolism and reverse cholesterol transport and it has been assumed that these systems contain large numbers of molecules whose overall behavior is deterministic, with stochasticity being negligible. Therefore to date it has largely been assumed that a stochastic approach would not present significant advantages. This does not mean however that we should completely ignore the possibility that stochasticity has a role to play in the dynamics of lipid metabolism. Regardless of the mathematical framework that is adopted, the computer simulates the interactions between the entities which results in a graphical account of how the biochemical species interact over time. In Fig. 2 the steps involved in the model building process are outlined. The process should be integrated with wet-laboratory experimentation so that each stage informs the other. Model predictions then provide the direction for further experimentation.

## 5 Computational Models of Lipid Metabolism

A variety of different levels of abstraction have been used to represent lipid metabolism, from cellular models to whole body representations. Models can be divided into two broad categories: those that focus on fatty acid metabolism and those that deal with the dynamics of cholesterol regulation. A recent example of a cellular model of cholesterol biosynthesis regulation showed that, the down-regulation of enzyme activity elicited a graduated reduction in flux along the pathway [106]. In contrast, modeling pharmacological interventions resulted in a similar degree of down-regulation in cholesterol synthesis, in a step change manner [106]. The authors suggest that the coordinate regulation of this pathway demonstrates a long-term evolutionary advantage over single enzyme regulation. Models that focus on lipoprotein dynamics have also been the focus of recent attention. Hübner et al. (2008) developed an interesting model of lipoprotein dynamics which was able to represent the lipoprotein profiles of healthy subjects and revealed heterogeneous lipoprotein distributions within the lipoprotein sub-fractions and predicted changes in lipoprotein distribution as a result of disease [107]. More recently the lead author of this article was involved in building a whole-body systems model of cholesterol metabolism that investigated the interaction of cholesterol metabolism with aging. The model was able to show that alterations to the absorption of intestinal cholesterol due to intrinsic aging could result in a significant increase in LDL-C [108]. The model also showed that decreasing the rate of hepatic clearance of LDL-C from half its initial value by age 65 years can result in the significant rise in LDL-C [108]. These findings helped to confirm that age-related alterations to key regulators of cholesterol metabolism have implications for CVD risk and suggested areas for potential therapeutic intervention. In a similar fashion van de Pas et al. (2012) developed a whole-body model of plasma cholesterol concentrations in humans [109]. This physiologically based pharmacokinetic model was adapted from a previous model designed for mice studies. The model was validated by comparing model predictions on plasma cholesterol levels of subjects with different genetic mutations with experimental data. Average model predictions on total cholesterol were accurate within 36% of the experimental data. A sensitivity analysis suggested that HDL-C concentration was mainly dependent on hepatic transport of cholesterol to HDL, cholesterol ester transfer from HDL to non-HDL, and hepatic uptake of cholesterol from non-HDL-C. Lu et al. (2014) focused specifically on RCT and constructed an *in silico* model of this process [110]. This ODE model was calibrated using a virtual population, and was used to explore potential ways of raising HDL-C. Intriguingly, the model suggested that CETP inhibition would not result in an increased RCT rate, contradicting the proposed role of CETP inhibition as a therapeutic target for improving RCT [111]. Recent models focusing on fatty acid metabolism include a model by Micheloni et al. (2014) which includes the key pathways of an adipocyte [112]. VLDL assembly in human liver has also been represented computationally [113]. This model was able to predict the plasma FFA composition required to generate a given alteration in the fatty acid composition of a lipoprotein. It is important to emphasize that regardless of the nature of the model, it can be used to examine the interaction of aging with lipid metabolism. This simply involves adjusting the parameters to reflect the impact of aging on a ‘normal’ biological system. Therefore in theory any of the computational systems models that have been discussed are capable of doing this.

## 6 Integrating Lipid Metabolism With Other Metabolic Systems

It is clear age related changes to a wide range of biological systems significantly impact the dynamics of lipid metabolism. Therefore, models of lipid metabolism should be as holistic as possible to account for the wide range of factors that can impact its behavior. For instance, an integrated whole body model of cholesterol metabolism together with fatty acid metabolism would certainly be worthwhile developing in the near future. Moreover, it would be worthwhile developing a model that captures the interplay of lipid metabolism with whole-body insulin–glucose dynamics. Model integration has been facilitated by the advent of a number of frameworks that have been developed specifically for the representation of biological models [114]. These frameworks allow models to be shared and reused by researchers even if they do not use the same modeling software tool. Presently, the leading exchange format is the systems biology markup language [115]. Models that have been encoded in this framework can be archived in the BioModels database, a repository designed specifically for housing SBML models [116]. We searched the BioModels database with a specific focus on holistic/physiological models. It was discovered that BioModels contains a number of models of insulin–glucose dynamics. It would be worthwhile using the integrated model of glucose and insulin regulation (MODEL1112110004 [117]) to create a holistic physiological model of these systems. Another model that was identified which could equally be used is a hierarchical whole-body model of insulin signaling and glucose homeostasis (BIOMD0000000372 [118]). The major advantage of such models is that they have been encoded in SBML, which aids their future manipulation and potential assimilation within a lipid model. The aforementioned integrated models could be used to address the important question of the crosstalk between hepatic TG levels and insulin sensitivity, as recently increased circulating lipid levels have been associated with insulin resistance [119]. Insulin resistance is the result of an inability to suppress hepatic glucose production or to stimulate peripheral glucose uptake. It is not clear if the increased production of hepatic TG affects insulin sensitivity, therefore this would be a worthwhile area for a combined model to investigate. It would also be useful to include the effects of genetic polymorphisms associated with lipid metabolism. For example, the presence of two defective alleles in the gene locus of LPL results in a significant loss in the activity of LPL and causes hypertriacylglycerolemia [120]. Thus, it is logical that examining such perturbations would present an additional insight into how a variety of mechanisms affect lipid metabolism. Combined lipid-stress hormone models are also needed, as chronic exposure to stress impacts cardiovascular health. Recent findings suggest that the dysregulation of cortisol homeostasis is a notable contributor to CVD [121]. This could be the result of a number of interactions. For instance, cortisol abnormalities are associated with increased abdominal obesity [122]. This suggests that alterations to adipose tissue are dependent on an increase in insulin, provoked by cortisol excess. Additionally it is suggested that obesity, with a disproportional increase of visceral fat depots, is a condition associated with elevated cortisol secretion [123]. These factors need to be investigated and it would be worthwhile applying a combined cortisol–lipid model to do this. The construction of this combined model could be facilitated by using one or a combination of a number of pre-existing models of cortisol homeostasis [124], [125], [126]. However, it is important to emphasize that multi-scale modeling is not straight forward, as the challenge of representing spatial and temporal scale has yet to be fully resolved. Moreover combining models of different scales is not a trivial task as one needs to consider different time scales and different levels of detail which invariability characterize each model. In our opinion it is necessary to focus initially on combining models of biological systems whose behavior has been well characterized. The issue of model parameterization also needs to be considered, as the larger the model becomes the more parameters it has. Furthermore, it is important to emphasize that even if two models are coded in SBML difficulties persist as inconsistencies often exist in the nomenclature of species and reactions from one model to another. A proposed solution to this difficulty is to use, The Minimal Information Required in the Annotation of Models (MIRIAM) project, which has the goal of generating a set of guidelines for the standard annotation and curation of computational models in biology [127]. It is suitable for use with any structured format for computational models. In the penultimate section we will explore these issues in more detail and present a number of models that have endeavored to overcome these challenges.

## 7 Obstacles and Opportunities for the Future

The impact of aging on lipid metabolism is complicated by a number of factors. If we take cholesterol metabolism as an example, it is known that LDL-C rises significantly with age (by ~ 40% between the 20th and 60th decade) [73]. Accounting for this rise should be the goal of any successful computational model of cholesterol metabolism. However, achieving this objective is hampered to a degree by the large number of factors that we have discussed that play a role in the rise of LDL-C. This issue is further complicated by the fact that there are sex-related differences in the development of hypercholesterolemia with age (reviewed in [128]). This issue adds another dimension to modeling lipid metabolism, for example is it feasible to integrate the key components of lipid metabolism with other biological systems such as cortisol or estrogen regulation. Another challenge centers on individual heterogeneity. As an example it is known that cholesterol absorption can vary from ~ 29% to ~ 80% [46]. Moreover, recent findings suggest that individual differences in cholesterol synthesis are an important consideration also [129]. These are factors that should be considered when designing a model of lipid metabolism. Regardless of the factors that are selected for inclusion in a model of lipid metabolism assumptions will remain a necessity, as the mathematics that underpins the biology has not always been fully delineated. In the absence of a detailed mathematical description, alternative approaches could be used. For instance, several recent models have successfully adopted constraint based modeling, a technique that represents a biological system by using a series of constraints, which characterizes its possible dynamics, facilitating a mechanistic description of metabolic physiology [130]. This approach could be used to help investigate the metabolic behavior of human cells and tissues to help unravel the mechanisms that underlie the changes to lipid metabolism with age. By introducing a successive series of constraints, an idea of the behavior of cells, tissues and organs during a range of scenarios could be obtained, thereby improving the available information to determine the underlying mechanisms [130]. This approach was recently successfully applied to lipid metabolism by Galhardo and colleagues (2014) to integratively study data from human adipogenesis, together with data on gene expression, genome-wide ChIP-seq profiles for peroxisome proliferator-activated receptor (PPAR), a nuclear receptor that regulates fatty acid storage, along with a variety of other information including microRNA data [131]. Another method that has been employed to integrate diverse data sets was utilized by Gupta and colleagues (2011) when they integrated metabolomics and transcriptomics data with legacy knowledge to create a worthwhile model of the sphigolipid pathway [132]. A further way to alleviate the problem would be to formulate experimentally testable hypotheses based on the *in silico* analysis of a well experimentally characterized regulatory circuit, perform the experiments, and update the models based on the ‘true’ predictions. This type of approach is ubiquitously employed to help understand the underlying regulatory networks in microbial metabolism [133]. This would undoubtedly lead to a better understanding of the mechanisms embedded within the systems and ultimately to an improvement of models of this nature. If this approach was adopted for lipid metabolism the challenge would be to determine which components to work with. Lipid absorption would be difficult as it remains poorly understood, whereas, cholesterol biosynthesis may be a suitable starting place as it has been well characterized. The recent models of cholesterol biosynthesis by Bhattacharya et al. (2014) [134] and the model by Mazein and colleagues (2013) are both suitable starting points [135]. The latter model is particularly useful, as it has been encoded in systems biology graphical notation (SBGN), an emerging standard for the graphical representation of systems biology models [136].

If the mathematics that underpins a well characterized component of lipid metabolism is established, it is then necessary to obtain suitable model parameters. Parameter uncertainty is currently an issue for mathematically modeling lipid metabolism and remains an issue for the field generally. The problem of parameter uncertainty was emphasized recently when a number of systems models within the BioModels database were investigated and found to have issues with their sensitivities [137]. For kinetic based models the velocity of a reaction in a systems model is generally described by a rate equation that typically assumes mass action kinetics or is based on an enzyme kinetic law (e.g. Michaelis–Menten kinetics). There are several databases which archive kinetic data [138], [139], [140], although the parameters they archive can vary significantly depending on the circumstance in which their kinetics was quantified. Obtaining human in vivo kinetic data is challenging due to the difficulties associated with measuring these parameters. One way to overcome this problem may be to quantify enzyme kinetics under carefully controlled in-vivo like conditions [141], [142].

The ultimate test of any computational model is its ability to provide additional insight into a complex problem, and effective parameter inference is central to achieving this. Recently, a broad range of statistical approaches has been applied to this field [143], [144], [145], [146], [147]. The final challenge is one of scale. In order for computer models to accurately capture the dynamics of lipid metabolism and aging in the future it will be necessary for them to be multi-scaled in nature. This means that to be an accurate representation of biological reality systems models need to incorporate the circuitry between differing spatial and temporal scales. For lipid metabolism this means being able to connect a model of intracellular cholesterol homeostasis/fatty acid metabolism all the way through to the physiological behavior of lipoproteins and their interactions. Overcoming this problem is an enormous challenge for the future of computational modeling in this area. Recently, a tentative step towards representing multi-scale biochemical relationships was developed by Karr and colleagues (2012) who created a “whole cell” model of the bacterium *Mycoplasma genitalium* [148]. The total functionality of the cell was divided into twenty eight sub-models. Each sub-model was modeled independently and then integrated with the other models. The model provided insights into cellular behavior, including in vivo rates of protein-DNA association and DNA replication. It is possible that an approach similar to this could be used to model lipid metabolism in a multi-scale manner in the future. Such a comprehensive model could provide the groundwork for interventions which extend health-span by delaying the onset of age related diseases such as CVD.

## 8 Conclusions

The aging process is characterized by changes affecting all aspects of an organism and results in an increased vulnerability to disease with time. The dysregulation of lipid metabolism has a long standing relationship with cardiovascular disease; the main cause of both morbidity and mortality in older people. To study the dynamics of lipid metabolism, computational models are becoming increasing used. The goal is to use the models to represent the dynamics of lipid metabolism in a quantitative fashion which is capable of encapsulating the key mechanisms of this complex system. In the future it is important to use these models to investigate how age-related changes to other biological systems impinge on lipid metabolism. For example, a plethora of hormonal fluctuations has an impact on lipid metabolism; therefore it is imperative they are included in future models. A major limitation of computational models of lipid metabolism is that there is a significant disparity between the current biological understanding and how the biological mechanisms are described mathematically, thus there is an urgent experimental need to characterize the biological mechanisms further. Another challenge for computational modeling this area centers on determining suitable parameters that capture the ‘true’ dynamics of lipid metabolism. Recent advances in this area have witnessed efforts to suitably quantify enzyme kinetics and have also focused on using techniques such as constraint based modeling. Overcoming these challenges is important for the future progress of computational models that represent the dynamics of lipid metabolism and for the development of novel strategies which augment health-span.

## Author Contributions

Mark Mc Auley conceived the idea for this review. Kathleen Mooney contributed to the nutrient focused sections.

## Competing Interests

The authors declare no conflict of interest.
